# Violence across the Life Course and Implications for Intervention Design: Findings from the Maisha Fiti Study with Female Sex Workers in Nairobi, Kenya

**DOI:** 10.3390/ijerph20116046

**Published:** 2023-06-03

**Authors:** Tara S. Beattie, Rhoda Kabuti, Alicja Beksinska, Hellen Babu, Mary Kung’u, The Maisha Fiti Study Champions, Pooja Shah, Emily Nyariki, Chrispo Nyamweya, Monica Okumu, Anne Mahero, Pauline Ngurukiri, Zaina Jama, Erastus Irungu, Wendy Adhiambo, Peter Muthoga, Rupert Kaul, Janet Seeley, Helen A. Weiss, Joshua Kimani

**Affiliations:** 1 Department of Global Health and Development, London School of Hygiene and Tropical Medicine, London WC1H 9SH, UK; 2 Partners for Health and Development in Africa, Nairobi P.O. Box 3737-00506, Kenya; 3 Department of Medicine, University of Toronto, Toronto, ON M5S 1A8, Canada; 4 MRC International Statistics and Epidemiology Group, Department of Infectious Disease Epidemiology, London School of Hygiene and Tropical Medicine, London WC1E 7HT, UK

**Keywords:** violence, female sex workers, Kenya, HIV, adverse childhood experiences (ACEs)

## Abstract

We examined violence experiences among Female Sex Workers (FSWs) in Nairobi, Kenya, and how these relate to HIV risk using a life course perspective. Baseline behavioural–biological surveys were conducted with 1003 FSWs June-December 2019. Multivariable logistic regression models were used to estimate the adjusted odds ratio (AOR) and 95% confidence intervals (CI) for associations of life course factors with reported experience of physical or sexual violence in the past 6 months. We found substantial overlap between violence in childhood, and recent intimate and non-intimate partner violence in adulthood, with 86.9% reporting one or more types of violence and 18.7% reporting all three. Recent physical or sexual violence (64.9%) was independently associated with life course factors, including a high WHO Adverse Childhood Experiences (ACE) score (AOR = 7.92; 95% CI:4.93–12.74) and forced sexual debut (AOR = 1.97; 95% CI:1.18–3.29), as well as having an intimate partner (AOR = 1.67; 95% CI:1.25–2.23), not having an additional income to sex work (AOR = 1.54; 95% CI:1.15–2.05), having four or more dependents (AOR = 1.52; 95% CI:0.98–2.34), recent hunger (AOR = 1.39; 95% CI:1.01–1.92), police arrest in the past 6 months (AOR = 2.40; 95% CI:1.71–3.39), condomless last sex (AOR = 1.46; 95% CI:1.02–2.09), and harmful alcohol use (AOR = 3.34; 95% CI:1.74–6.42). Interventions that focus on violence prevention during childhood and adolescence should help prevent future adverse trajectories, including violence experience and HIV acquisition.

## 1. Introduction

Violence against women and girls (VAWG) is common around the world, with almost one in three women (31%) experiencing physical or sexual violence (PSV) against them by an intimate partner or sexual violence from someone other than their partner in their lifetime [1]. Intimate partner violence (IPV) prevalence varies substantially between countries and in different geographical locations within countries [2]. In Kenya, the 2014 Demographic and Health Survey estimated that 47% of women aged 15–49 years have ever experienced PSV from a spouse or someone else, with 20% reporting physical violence and 8% reporting sexual violence in the previous 12 months [3]. Although IPV is the most common form of violence experienced, VAWG is multi-faceted, and women can experience violence from different perpetrators (e.g., family members, strangers, etc.) and in different forms (e.g., childhood sexual abuse, forced sexual debut, physical, emotional and/or sexual IPV, elder violence) across their life course [1]. For both women and men, experiences in childhood, such as experiencing violence or witnessing violence against their mother is associated with increased risk of experiencing or perpetrating violence in adulthood [4,5,6,7]. In addition, men who are violent towards women often have a clustering of risk behaviours that can increase the chances that they and/or their sexual partners will acquire HIV and other STIs [4,8,9,10]. At least three prospective cohort studies from sub-Saharan Africa (SSA) confirm that physical IPV and any IPV are associated with increased HIV incidence [11].

Female sex workers (FSWs) are at increased risk of both violence and HIV compared to all women [12,13]. A systematic review found that the prevalence of workplace violence only (i.e., not including IPV) was 45–75% ever and 32–55% in the past year [14]. In addition, the relative risk of acquiring HIV is 30 times greater among FSWs compared with women of reproductive age from the general population [15]. The criminalisation of sex work in most settings globally means that FSWs work on the margins of society and legality, with little recourse for violence against them, meaning perpetrators can continue with impunity [16]. Police officers and other law enforcement agents frequently perpetrate instead of protect FSWs from violence, including through police arrest and imprisonment, as well as demanding sex in exchange for non-arrest [14,17,18]. FSWs have multiple identities, including as daughters, wives/intimate partners, mothers, and key providers for their families [19]. Thus, in addition to ‘workplace’ violence, they are also at risk of childhood, family, partner, and other non-sex-work related violence [20,21]. However, little is known about the co-occurrence of violence across the life course among FSWs. In addition, to our knowledge, there have been no studies to date that have examined violence exposure among the children of FSWs. Together these have important implications for the ‘transfer’ of violence between generations and the need for interventions.

Kenya is located in East Africa and has one of the largest numbers of people living with HIV globally. The adult HIV prevalence is estimated to be 4.0% and is higher in women (5.4%) compared with men (2.6%) [22]. Nairobi is the capital and largest city in Kenya, with a population of approximately 4.4 million people [23]. Nairobi county has an estimated 2032 ‘hot-spots’ where approximately 39,600 women sell sex [24]. Types of hot-spots include bars with lodging (where sex work can take place), bars without lodging, guest houses, streets, sex dens, and uninhabited buildings. Around 73% (29,000) of FSWs in Nairobi are served by seven Sex Worker Outreach Programme (SWOP) clinics which provide peer education and outreach, comprehensive clinical services, including HIV testing and treatment, and condom distribution. Additional programmes provide services for other FSWs. The Maisha Fiti study is a mixed-methods longitudinal study which aims to examine the biological impact of violence and harmful drinking on inflammation in the blood and the genital tract. Using baseline data collected from June–December 2019, the aim of this paper is to examine (i) violence experiences across the life course and (ii) associations of risk factors across the life course with recent violence experiences among FSWs in Nairobi.

## 2. Materials and Methods

### 2.1. Study Design and Sampling

The Maisha Fiti study was designed in consultation with the FSW community in Nairobi, as well as with peer educators and staff working at the seven SWOP clinics. The study was powered to detect genital inflammation among women who had experienced recent PSV. Assuming 2:1 exposure to recent violence, enrolling 750 HIV-negative women would detect a 10% absolute difference in the proportion of women who have genital inflammation (25% vs. 15%) at 90% power. The HIV prevalence among FSWs in Nairobi is approximately 25%, and thus, the target sample size was 1000 FSWs for the study.

All women attending SWOP clinics have a unique enrolment number supported by biometrics (fingerprints). Enrolment numbers were selected from all clinic attendees who had accessed SWOP services in the past 12 months, who were aged 18–45 years, and who did not have an underlying chronic illness (other than HIV) that was likely to alter host immunology. Of 29,000 FSWs enrolled at one of the seven SWOP clinics across Nairobi, 10,292 met these inclusion criteria and were included in the sampling frame. Additional exclusion criteria (assessed during study enrolment) were current pregnancy or breastfeeding. Of the 10,292 FSWs, 1200 were randomly selected for study participation with numbers weighted by the total population of FSWs enrolled in each SWOP clinic. Women aged <25 years were oversampled to enable sufficient power for analyses stratified by age. Thus, although <25 year olds represented 11.69% of women meeting the study inclusion criteria, we randomly selected 21.14% to participate in the study (sampling fraction: <25 year olds 17.6%; 25+ years 8.7%).

### 2.2. Ethics and Informed Consent

The Maisha Fiti study was ethically approved by the Kenyatta National Hospital—University of Nairobi Ethics Review Committee (KNH ERC P778/11/2018), the Research Ethics Committees at the London School of Hygiene and Tropical Medicine (Approval number: 16229) and the University of Toronto (Approval number: 37046). Selected women were telephoned, informed about the study, and invited to the study clinic, where the study team gave them detailed study information both verbally and through a written participant information leaflet. Information about the study was also relayed to the sex work community by seven peer educators (The Maisha Fiti Study Champions). Consenting women undertook a pregnancy test, and those who were not pregnant or breastfeeding were enrolled in the study and completed a behavioural–biological survey. Those found to have experienced recent violence or to have mental health problems or suicidal behaviours were referred to a trained counsellor employed as part of the study team. All women who tested positive for HIV were counselled and referred for HIV care at their SWOP clinic. All women who tested positive for bacterial STIs were offered treatment free of charge.

### 2.3. Behavioural–Biological Survey

Women completed a baseline questionnaire focused on socio-demographics, sexual history and practices, reproductive health, Adverse Childhood Experiences (ACEs), sex work characteristics, intra-vaginal washing practices, mental health problems, and alcohol and substance use. The WHO Violence Against Women 13-item questionnaire, which measures the frequency and severity of Intimate Partner Violence (IPV), was adapted to include violence by non-IPs (e.g., clients, police, strangers etc.) [2]. We asked about lifetime violence experiences and those in the past 6 months. Following consultation with the sex work community, we also asked about (i) drugging and imprisonment (locked up somewhere against their will) by IPs and non-IPs, (ii) experiences of gang rape (ever and in the past 6 months), (iii) rape in the past 7 days, (iv) police arrest and imprisonment ever and in the past 6 months, and (v) if anyone had protected them from violence in the past 6 months.

**Figure 1:**
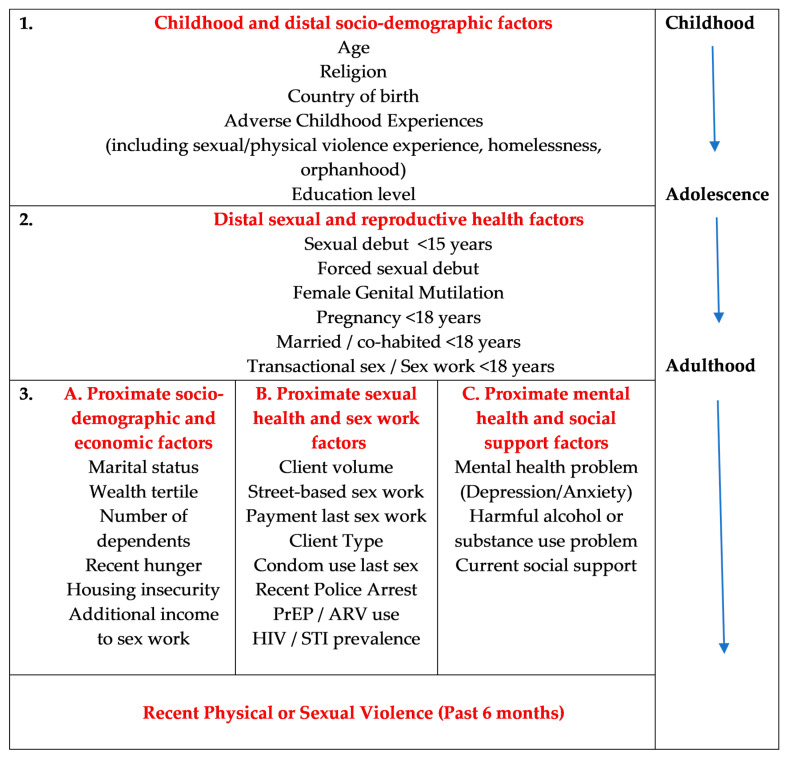
Conceptual hierarchical framework of risk factors across the life course for recent physical or sexual violence experience among FSWs.

Our main outcome variable was defined as physical and/or sexual violence in the past 6 months, as PSV is the category that had been the basis of most violence research [6,25]. We examined recent physical/sexual violence by anyone and by type of perpetrator (IP, non-IP). Exposure variables are shown in Figure 1, with further details provided in Table 1.

### 2.4. Laboratory Methods

Urine samples were collected to test for pregnancy, Chlamydia trachomatis (CT), and Neisseria Gonorrhoea (NG) infection. Blood was taken to test for Treponema pallidum (syphilis). HIV status was screened by rapid HIV tests, with positive tests confirmed using HIV DNA Genexpert. Self-collected vaginal swabs were used to test for Bacterial Vaginosis (BV; Gram’s stain and Nugent scoring) and Trichomonas vaginalis (TV; OSOM Trichomonas Rapid Test; SEKISUI Diagnostics, Massachusetts, USA).

### 2.5. Conceptual Framework

We developed a conceptual framework (Figure 1) based on a life course perspective [32] to explore the associations of recent physical or sexual violence with distal and proximate exposure variables, drawing on current theories about risk factors and drivers of violence [4,33]. Level 1 variables included ACEs and distal socio-demographic factors. Level 2 variables included distal sexual and reproductive health factors. Level 3 variables included (a) proximate socio-demographic and economic factors; (b) proximate sexual health and sex work characteristics; and (c) proximate mental health and social support factors.

### 2.6. Statistical Analyses

Data were double-entered using CSPro Software (United States Census Bureau, https://www.census.gov/data/software/cspro.html, (accessed on 1 June 2023)) and statistical analyses were conducted in STATA 16.1 (Stata Inc., College Station, TX, USA). We used a hierarchical modelling approach to build multivariable models for each outcome (recent physical/sexual violence by (i) any perpetrator; (ii) an intimate partner; and (iii) a non-intimate partner). Associations were estimated using odds ratios (OR), with *p*-values obtained using a joint hypothesis via the adjusted Wald test (to allow for sampling weights). Tests for trends were conducted for ordered categorical variables included in the final models. All models were adjusted for age and clinic as a priori defined variables. Level 1 variables associated with recent physical or sexual violence (*p*-value < 0.1) in univariate analyses were included in an initial multivariable logistic regression model. Variables were retained in a core Level 1 model (Model 1) if independently associated with any of the three violence outcomes (*p*-value < 0.1). Next, Level 2 variables were examined and adjusted for the core Level 1 variables and were retained if independently associated (*p*-value < 0.1) with any of the three violent outcomes (Model 2). Similarly, Models 3a–3c were fitted with Level 3a–3c variables, respectively, adjusting for core Level 1 and Level 2 variables. Missing data was reported if >5% of observations were missing.

## 3. Results

### 3.1. Sample Demographics and Sex Work Characteristics

Of 1200 sampled women, 1039 met the eligibility criteria, and 1003 (96.5%) consented to participate in the study. Participant characteristics are shown in Table 2. 64.9% of study participants had experienced recent (past 6 months) physical or sexual violence from any perpetrator (intimate or non-intimate partner). The median age of participants was 32 years (range 18–45 years). Most were born in Kenya (98.7%), were Catholic or Protestant (91.3%), and had primary education or less (70.1%). The median number of Adverse Childhood Experiences (ACEs) reported was 6 (range 0–12). The median age at sexual debut was 16 years (range 0–26 years), with one-third of women (31.2%) reporting their sexual debut was not consensual. Just over half of participants (59.8%) reported a current intimate partner (IP) (defined as a lover or boyfriend who does not pay for sex), although only 6.8% were living with a male partner. The most common places for soliciting clients were bars, clubs, or lodges (61.5%) or on the streets (30.0%), and the median number of clients in the previous week was 3 (range 0–70). HIV prevalence was 28.0%, but bacterial STI prevalence was relatively low (10.2%) (CT 5.7%; NG 2.6%; Syphilis 2.1%) (Table 2).

### 3.2. Violence Experience across the Life-Course

**Figure 2:**
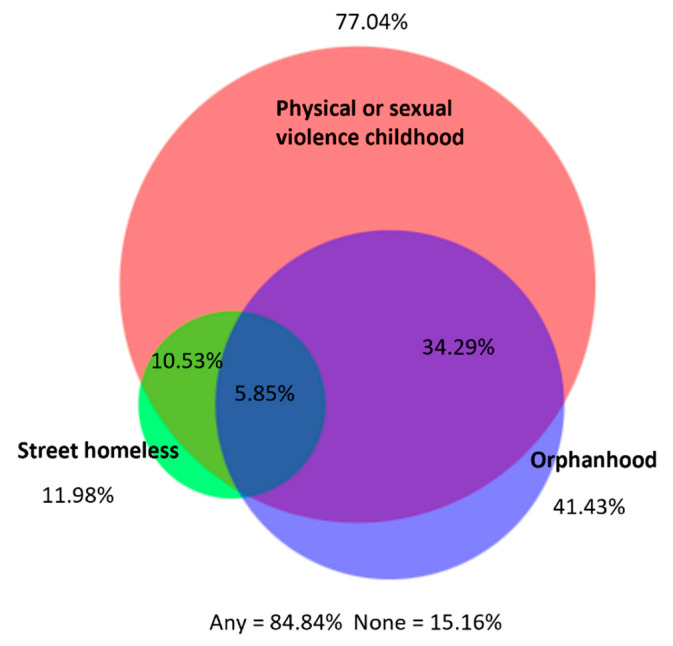
Overlap between physical/sexual violence, orphanhood, and street homelessness during childhood (<18 years old).

Reported ACEs were prevalent among study participants: 41.4% were orphaned, 12.0% had lived on the streets, 77.0% had experienced physical or sexual violence, and 89.9% had experienced war or community violence when they were a child (<18 years old). A small minority (6.3%) reported Female Genital Mutilation (FGM), and 11.6% reported being raped during their sexual debut (Table 3). There was substantial overlap between experiencing PSV, orphanhood, and street homelessness in childhood, with 84.84% reporting at least one of these events and 5.85% reporting all three (Figure 2).

Reports of recent violence experience were also common, with 64.9% experiencing PSV by any perpetrator, 31.2% of women experiencing PSV by an IP, and 55.7% experiencing PSV by someone other than an IP in the past 6 months (Table 3). In addition, participants reported being drugged or imprisoned by partners (6.9%) and non-partners (18.5%) and arrested by the police because they were sex workers (30.7%) in the past 6 months. A substantial minority (*n* = 61; 6.4%) reported being raped in the 7 days prior to the survey (Table 3). Of note, just over one-third of participants (35.9%) said that someone had protected them from violence in the past 6 months, including the police (15.3%), city askaris (3.2%), clients (14.1%) and other FSWs (5.9%) (data not shown).

**Figure 3:**
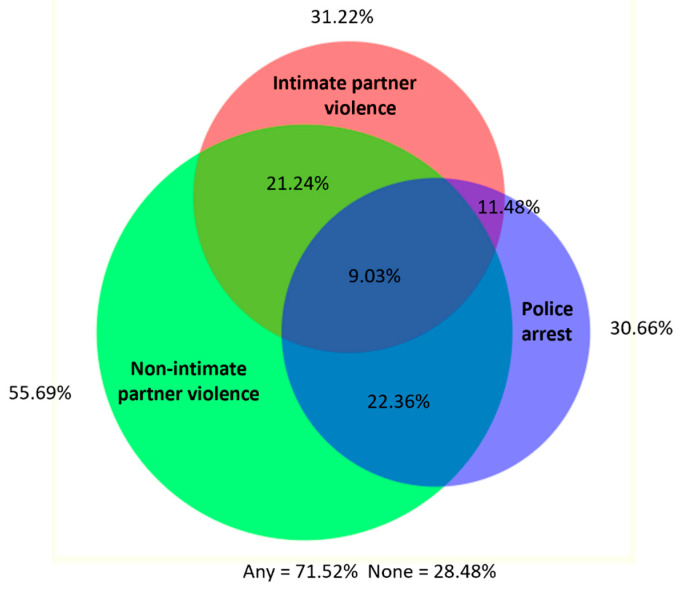
Overlap between recent (past 6 months) physical/sexual violence by an intimate partner, physical/sexual violence by a non-intimate partner and police arrest.

**Figure 4:**
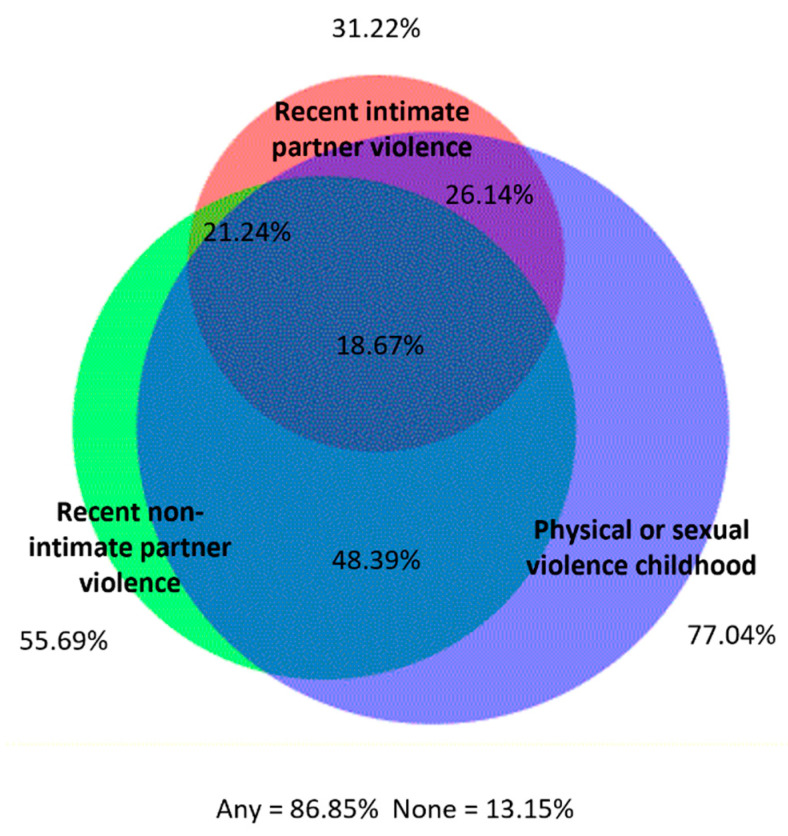
Overlap between physical/sexual violence during childhood (<18 years old) and recent (past 6 months) physical/sexual violence by an intimate partner and a non-intimate partner.

When we explored experiences of violence polyvictimization during the past 6 months, we found substantial overlap between recent physical or sexual violence by intimate partners, non-intimate partners, and police arrest, with 71.52% of women reporting any of these events and 9.03% reporting all three (Figure 3). Importantly, we also found substantial overlap between recent intimate and non-IP PSV and PSV experiences in childhood, with 86.85% reporting one or more types of violence and 18.67% reporting all three (Figure 4).

### 3.3. Witnessing Violence by Children of FSWs

We next investigated the violence experiences of children of FSWs, as reported by FSWs. Of the 911 participants who had children, 33.5% said their children had witnessed violence against them, with 18.1% witnessing violence against them in the past 6 months (Table 4). In addition, of the 55 participants who had children and who had been imprisoned in the past 6 months, 14.5% said that they had no one to look after their children during their imprisonment (data not shown).

### 3.4. Associations with Recent Physical or Sexual Violence Experience by Any Perpetrator

We were interested to understand factors across the life course which were associated with recent PSV experience by any perpetrator, guided by our conceptual framework (Figure 1). In Model 1 (Table 5), women with recent PSV experience by any perpetrator had a higher prevalence of ACEs (5–8 vs. 0–4 ACEs: AOR 3.01, 95% CI 2.23–4.07; 9–12 vs. 0–4 ACEs: AOR 7.92, 95% CI 4.93–12.74) and were more likely to be aged 25–34 years (rather than younger and older women) (Table 5, Model 1). In Model 2, after adjusting for level 1 factors, there was strong evidence that recent PSV was associated with a forced sexual debut (AOR 1.97, 95% CI 1.18–3.29). In Model 3a, after adjusting for level 1 and level 2 factors, participants with recent PSV experience by any perpetrator were more likely to currently have an IP (AOR 1.67, 95% CI 1.25–2.23), less likely to have an additional income to sex work (AOR 0.65, 95% CI 0.48–0.87) and were more likely to report recent hunger in the past 7 days (AOR 1.39, 95% CI 1.01–1.92), compared to women with no recent PSV experience. There was some evidence that having four or more dependents was also associated with an increased risk of recent violence (AOR 1.52, 95% CI 0.98–2.34) (Table 5, Model 3a). When we examined associations with proximate sexual health and sex work characteristics (Model 3b), after adjusting for level 1 and level 2 factors, recent police arrest (AOR 2.40, 95% CI 1.71–3.39) and not using a condom at last sex (AOR 0.68, 95% CI 0.48–0.98) were associated with recent PSV by any perpetrator (Table 5, Model 3b). When we examined associations with proximate mental health and social support factors (Model 3c), after adjusting for level 1 and level 2 factors, we found that high alcohol risk score (AOR 3.34, 95% CI 1.74–6.42) and CBO membership (AOR 1.91, 95% CI 1.14–3.21) were associated with recent PSV by any perpetrator (Table 5, Model 3c).

### 3.5. Associations with Recent Physical or Sexual Violence Experience by an Intimate Partner

We next examined associations with recent PSV experience by an IP, with some similar findings to recent PSV by any perpetrator (Supplementary Materials: Table S1). Thus, recent PSV experience by an IP was associated with a high number of ACEs (Model 1), having an intimate partner (Model 3a), not using a condom at last sex and recent police arrest (Model 3b), and a moderate or high alcohol use score (Model 3c). In addition, there was evidence that having an additional income from sex work (AOR 1.32, 95% CI 0.97–1.78) (Model 3a), non-use of PrEP, PEP or ARV (AOR 0.67, 95% CI 0.50–0.89) (Model 3b), and mild (AOR 2.22, 95% CI 1.58–3.12) or moderate/severe (AOR 1.40, 95% CI 0.95–2.04) depression or anxiety (Model 3c) were also associated with recent PSV experience by an IP—findings not seen when examining associations with recent PSV by any perpetrator. Conversely, there was no evidence that recent PSV experience by an IP was associated with the economic factors seen in the overall analyses (number of people dependent on her income, recent hunger, not having an additional income for sex work).

### 3.6. Associations with Recent Physical or Sexual Violence Experience by a Non-Intimate Partner

Associations with recent PSV by a non-intimate partner were broadly similar to recent PSV by any perpetrator (Supplementary Materials: Table S2), with street-based sex work additionally associated with increased risk of recent PSV by a non-intimate partner (AOR 1.48, 95% CI 1.08–2.01) (model 3b).

## 4. Discussion

We found a high prevalence of recent physical or sexual violence experiences among FSWs in Nairobi, with 64.9% of women reporting PSV by any perpetrator in the previous six months. Key risk factors during childhood and adolescence include a higher prevalence of ACEs and forced sexual debut. More proximate risk factors include having four or more dependents on her income, having an intimate partner, not having an additional income other than sex work, recent hunger, police arrest in the past 6 months, non-condom use at last sex, moderate or high alcohol use risk score, and belonging to an FSW CBO. We also found a high prevalence of PSV experience in childhood (77.0%) and a substantial overlap with recent intimate and non-IP violence in adulthood. This suggests an explicit need to understand violence against FSWs from a life course and intersectional perspective [34], as many FSWs are adversely situated economically, socially, and politically throughout their life course. Taken together, these findings provide strong evidence of polyvictimization, including sequential and concurrent violence experiences, findings which are supported by our qualitative research [35]. A key limitation of our study was the cross-sectional design, meaning the direction of causality cannot be ascertained; our findings and implications should be interpreted with this in mind.

The high prevalence of violence against FSWs that we found here has been described in multiple other settings. In a systematic review from 2014, the lifetime prevalence of workplace violence was 45–75%, and in the past year, workplace violence was 32–55% [14]. Previous studies with FSWs in Mombasa have reported similarly high violence levels, with 63% reporting lifetime physical violence and 44% reporting lifetime sexual violence [36]. Recent studies with FSWs in Russia, Rwanda, South Africa, and the USA have also found evidence of lifetime polyvictimization [21,37,38,39]. However, we found only one other study with FSWs from SSA, which examined recent violence (rape in the past year) from a life-course perspective [40]. The authors found that childhood trauma, food insecurity, and harmful drinking were similarly associated with recent sexual violence and street-based sex work was similarly associated with recent rape by a non-IP.

Among FSWs, the three pillars of a comprehensive GBV response include (i) prevention, (ii) survivor support, and (iii) accountability/justice [41]. A recent systematic review identified 21 FSW violence interventions in 10 countries [41]. Evidence from India suggests that a combination of HIV and workplace violence interventions can successfully impact both HIV and violence outcomes, including police arrest [42,43,44]. Mathematical modelling suggests that reducing violence against FSWs in Kenya (and Ukraine) would also significantly reduce HIV infections among FSWs and the general population [45]. SWOP introduced violence interventions and response mechanisms and reporting as part of its programming in Nairobi in 2014. In line with the national government violence prevention and response strategy for key populations, this included both violence prevention and violence response interventions [46]. Interestingly, proximal protective factors for reduced violence risk among FSWs in our study include financial (reduced number of people she supports on her income, having an additional income to sex work), workplace (not selling sex on the street), and individual (not having an intimate partner); findings supported by our qualitative research and also by FSW studies elsewhere [35]. However, there remains a paucity of evidence regarding effective combination interventions for IPV (i.e., spousal/partner violence) and HIV among key populations, despite the fact that key populations of women bear a disproportionate burden of both IPV and HIV.

It is well recognised that witnessing or experiencing violence in childhood is strongly associated with IPV in adulthood among all women [4,5,6]; our study adds to the literature on FSWs, which finds that ACEs are also associated with increased risk of recent PSV among FSWs, both by intimate partners and by others. Importantly, one-third of FSWs said their children had witnessed violence against them, providing evidence of the inter-generational transfer of violence experience. The WHO INSPIRE technical package has been developed to assist countries in developing programmes to support children exposed to trauma and to prevent violence against children [47]. Economic strengthening for families could also help reduce the risk of ACEs [48,49], as well as reduce the risk of subsequent entry to sex work for economic survival.

The strengths of our study include a large, random sample of FSWs from across Nairobi, the use of validated tools to measure key variables, and the use of biological samples to measure HIV and STIs. In addition, our qualitative interviews and public engagement workshops with study participants and peer educators helped support and interpret our study findings. As noted above, a key limitation was the cross-sectional design; longitudinal data will become available for this study shortly, although long-term cohort studies would be needed to causally associate events in childhood and adolescence with violence experienced during adulthood. Our sampling methodology missed an estimated 10,600 FSWs who are not registered at a SWOP clinic (although they may be registered at other non-SWOP services). These women may be more vulnerable and at increased risk of the study outcome and key exposures (such as harmful drinking and younger age), leading to an underestimation of violence prevalence. However, creating a sampling frame and randomly selecting from all FSWs working in Nairobi was prohibitively expensive for our study.

## 5. Conclusions

Levels of violence experienced by FSWs in Nairobi, Kenya, are extremely high and occur throughout their life course. This warrants interventions to both prevent violence—especially at pivotal periods in the life course (childhood, adolescence, first relationships)—as well as programming to support FSWs (and their children) who experience violence [50]. Preventing violence against FSWs would also help protect their children from violence exposure and thus help break the cycle of violence across the life course. Decriminalisation of sex work in Kenya and other countries and addressing violence perpetrated by men would be key steps in supporting this transition [51,52,53].

## Figures and Tables

**Table 1:** Definition of Exposure Variables used in the Maisha Fiti study with FSWs in Nairobi, Kenya.

Variable	Tool/Question	Category
Childhood and Distal Socio-Demographic Factors		
ACEs	WHO Adverse Childhood Experiences International Questionnaire (ACE-IQ) [26]	Individually and Ordered categorical variable (each ACE scores one point): <4, 5–8, 9–12 [27]
Distal Sexual and Reproductive Health Factors		
Forced sexual debut	Would you say you wanted to have sex that first time, or was it against your will?	I wanted to have sex vs. I was tricked into having sex, or I was pressured into having sex vs. I was physically forced to have sex
Female Genital Mutilation	Have you ever had a surgical procedure used for modifying the vagina or restoration of the hymen, including female genital circumcision, incision with insertion of substance into the lesion?	No vs. Yes
Age at first sex work	How old were you when you first received money/goods in exchange for sex?	<18, 18–24, 25–40 years
Proximate Socio-Demographic and Economic Factors		
Wealth Tertiles	14 household asset questions used in the Kenyan Demographic Health Surveys [3]	Principle component analysis (PCA) used to compute household wealth tertiles: Low, Medium, High
Number of dependents	Not including yourself, how many people living in your household are dependent on your income? How many people outside your household are dependent on your income?	0–1, 2–3, 4+ dependents
Recent hunger	Thinking now about the past 7 days, have you or anyone in your family skipped a meal because there was not enough food?	No vs. Yes
Proximate Sexual Health and Sex Work Factors		
Recent police arrest	Have you been arrested in the past 6 months because you are a sex worker?	No vs. Yes
Current PrEp/PEP/ARV use	Are you currently taking PrEP? (HIV negative) Are you currently taking PEP? (HIV negative) Are you currently taking ARVs? (HIV positive)	No vs. Yes
Proximate Mental Health, Alcohol and Substance Use and Social Support Factors		
Depression	Patient Health Questionnaire-9 (PHQ-9) [28,29]	none 0–4; mild 5–9; moderate-severe 10+
Anxiety	Generalised Anxiety Disorder-7 Assessment (GAD-7) [28,30]	None 0–4; mild 5–9; moderate-severe 10+
Harmful alcohol use	WHO ASSIST (Alcohol, Smoking and Substance Involvement Screening Test) tool [31]	Low risk 0–10; moderate risk ≥11; high risk >27
Harmful substance use	WHO ASSIST (Alcohol, Smoking and Substance Involvement Screening Test) tool [31]	Low risk 0–4; moderate risk >4; high risk >27
Current social support	Do you have someone who you can talk to about your problems?	Yes/Sometimes vs. No

**Table 2:** Socio-demographic and sex work characteristics of study participants.

		*n* 1003	%	% Physical or Sexual Violence by any Perpetrator Past 6 Months (*n* = 646)
Childhood and Distal Socio-Demographic Factors				
Age (years)	18–24	212 353 438	11.7 39.4 48.9	60.4 70.8 61.2
	25–34			
	35–45			
	Mean Age 33.7 (SD 7.1)			
Religion	Catholic	375	36.9	62.0
	Protestant	534	54.4	67.2
	Muslim	46	4.6	68.3
	Other/None	46	4.1	57.8
Country of birth	Kenya	989	98.7	65.2
	Other	14	1.3	45.8
Total number of Adverse Childhood Experiences *	0–4	282	28.4	43.1
	5–8	548	54.3	69.6
	9–12	173	17.3	85.8
Education Level	<Primary	169	17.8	63.0
	Primary only	525	52.3	65.5
	≥Secondary	309	29.9	64.9
Distal Sexual and Reproductive Health Factors				
Age at sexual debut (years)	<16	369	37.3	69.8
	16–17	312	30.0	66.2
	18+	315	32.7	58.1
Sexual debut forced	Consented	695	68.8	60.4
	Tricked/pressured	194	19.6	71.4
	Forced	110	11.6	80.2
Eldest child conceived when <18 years	No	517	52.4	63.1
	Yes	486	47.7	66.9
Married/co-habited < 18 years	No	759	75.8	64.0
	Yes	244	24.3	67.8
Age at first sex work	<18	176	16.6	71.2
	18–24	474	44.9	65.6
	25–40	341	38.5	61.6
**Proximate socio-demographic and economic Factors**				
Current Intimate Partner	No	392	40.2	59.6
	Yes	610	59.8	68.4
Current living status	Lives alone	139	13.8	53.6
	Lives with parents/friends	103	7.7	60.4
	Lives with children only	693	7.2	66.6
	Lives with partner (+/− children)	67	6.8	74.6
Number of children living with her	None	113	12.3	58.6
	1–2	558	64.7	66.4
	3+	193	23.0	71.3
Wealth Tertile	Low	334	32.6	71.4
	Medium	333	33.5	68.0
	High	333	34.0	55.4
Number of people dependent on her income	0–1	178	15.4	54.3
	2–3	475	48.6	63.9
	4+	350	36.0	70.7
Recent hunger past 7 days	No	670	66.1	59.6
	Yes	331	33.9	75.0
Additional income to sex work	No	571	56.4	68.4
	Yes	432	43.7	60.4
Proximate Sexual Health and Sex Work Factors				
Client volume past week	<5	607	60.9	62.4
	5–9	250	24.8	70.9
	10+	137	14.2	66.0
Place where solicit clients	Phone/internet	54	5.4	61.9
	Home/middle-men/markets	15	1.6	67.9
	Brothel/escort service/massage	14	1.5	53.9
	Bar/club/lodge	620	61.5	62.5
	Street/bus/taxi	294	30.0	71.5
Condom use last sex with a client	No	174	17.3	67.2
	Yes	819	82.7	64.5
Condom use last sex (any partner)	No	236	22.8	70.7
	Yes	765	77.2	63.1
HIV prevalence	Negative	746	72.0	65.6
	Positive	257	28.0	63.1
Bacterial STI prevalence **	None	891	89.8	64.9
	One+	112	10.2	65.2
Proximate Mental Health, Alcohol and Substance Use and Social Support factors				
Depression or Anxiety score (PHQ-9/GAD-7)	Low	481	47.1	58.2
	Mild	279	27.9	67.9
	Moderate/Severe	243	25.0	74.1
Alcohol risk score (WHO ASSIST)	Low 0–10	697	70.2	59.2
	Moderate 11–26	207	20.4	73.6
	High 27+	95	9.4	88.1
Substance *** risk score (WHO ASSIST)	Low	759	96.4	61.7
	Moderate	228	21.8	73.9
	High	18	1.8	90.7
Has someone can talk to	No	278	27.5	64.4
	Yes	725	72.5	65.1
Belongs to an FSW CBO	No	907	90.4	63.4
	Yes	96	9.6	78.6

* Total number of Adverse Childhood Experiences [26] defined as answering yes to:When you were growing up, during your first 18 years of life, (i) Did you live with a household member who was a problem drinker or alcoholic, or misused street or prescription drugs? (ii) Did you live with a household member who was depressed, mentally ill or suicidal? (iii) Did you live with a household member who was ever sent to jail or prison? (iv) Were your parents ever separated or divorced? (v) Did your mother, father or guardian die? (vi) Did you witness violence in the home (ACE 4.6, 4.7, 4.8) (vii) Did you experience emotional violence (ACE 5.1, 5.2) (viii) Did you experience physical violence (ACE 5.3, 5.4) (ix) Did you experience sexual violence (ACE 5.5, 5.6, 5.7, 5.8) (x) Did you experience community violence (ACE 7.1–7.3) (xi) (ACE 8.1–8.4) (xii) Did you ever live on the streets? ** Current bacterial STI prevalence defined as a positive test for gonorrhoea, chlamydia and/or syphilis infection. *** Substance risk score (WHO ASSIST) (excluding cigarettes and alcohol) [31] defined as “high” if participant’s highest score was “high” for any substance, “moderate” if participant’s highest score was “moderate” for any substance, and “low” if they scored “low” for all substances. Substances asked about included Cannabis, Cocaine, Amphetamine type stimulants, Inhalants, Sedatives or Sleeping Pills, Hallucinogens, and Opioids.

**Table 3:** Prevalence of violence experience across the life-course among FSWs in Nairobi, Kenya.

		*n*	%
Childhood violence experiences (<18 years old)	Witnessed physical abuse at home	707	70.7
	Experienced verbal abuse	609	61.3
	Experienced threats of or actual abandonment	433	43.5
	Experienced physical abuse	656	65.8
	Experienced sexual violence	460	46.2
	Experienced physical or sexual violence	769	77.0
	Witnessed community violence	896	88.7
	Experienced war/collective violence	353	34.3
	Experienced physical violence from soldiers, police, militia, or gangs	104	10.5
	Had a family member or friend who was beaten up or killed	192	19.5
	Experienced female genital cutting	60	6.3
	Experienced forced sexual debut	110	11.6
Violence experiences from any perpetrator (IP or non-IP)	Physical or sexual violence ever	811	81.6
	Emotional violence in the past 6 months	746	74.8
	Physical violence in the past 6 months	552	55.0
	Sexual violence in the past 6 months	499	50.6
	Sexual violence in the past 7 days	61	6.4
	Physical or sexual violence in the past 6 months	646	64.9
	Drugged or imprisoned in the past 6 months	221	22.1
Intimate partner violence (IPV) experiences	Physical or sexual violence ever	560	56.3
	Emotional violence in the past 6 months	302	29.5
	Physical violence in the past 6 months	254	25.2
	Sexual violence in the past 6 months	220	22.6
	Sexual violence in the past 7 days	44	4.6
	Physical or sexual violence in the past 6 months	313	31.2
	Drugged or imprisoned in the past 6 months	68	6.9
Violence experiences from others (non-IP)	Physical or sexual violence ever	688	69.2
	Emotional violence in the past 6 months	698	70.1
	Physical violence in the past 6 months	446	44.7
	Sexual violence in the past 6 months	397	40.1
	Gang rape in the past 6 months	23	2.4
	Sexual violence in the past 7 days	27	2.8
	Physical or sexual violence in the past 6 months	554	55.7
	Drugged or imprisoned by non-IP in the past 6 months	184	18.5
Police experiences	Arrested ever because of sex work	563	58.4
	Arrested in the past 6 months because of sex work	302	30.7
	Avoided arrest in the past 6 months using sex	67	6.8
	Imprisoned in the past 6 months because of sex work	61	6.5

**Table 4:** Prevalence of children <18 witnessing violence against their mother among FSWs in Nairobi, Kenya.

		*n* = 911 ^ *^	%
Children <18 years	Children witness IP violence ever	211	23.8
	Children witness non-IP violence ever	139	15.7
	Children witness IP or non-IP violence ever	297	33.5
	Children witness IP violence in the past 6 months	100	11.0
	Children witness non-IP violence in the past 6 months	89	10.1
	Children witness IP or non-IP violence in the past 6 months	163	18.1

* Analysis restricted to 911 women who have children.

**Table 5:** Multivariable logistic regression—associations with recent physical or sexual violence (any perpetrator) among FSWs in Nairobi, Kenya.

			*n* (% *)	Crude Odds Ratio (95% CI)	Adjusted Odds Ratio ** (95% CI)	*p*-Value ***
Model 1	Age (years)	18–24	212 (60.4)	Ref	Ref	0.3
		25–34	353 (70.8)	1.59 (1.12–2.26)	1.73 (1.18–2.53)	
		35–45	438 (61.2)	1.03 (0.74–1.44)	1.12 (0.78–1.62)	
	Total number of Adverse Childhood Experiences	0–4	282 (43.1)	Ref	Ref	<0.001
		5–8	548 (69.6)	3.02 (2.25–4.05)	3.01 (2.23–4.07)	
		9–12	173 (85.8)	7.98 (4.97–12.81)	7.92 (4.93–12.74)	
Model 2	Sexual debut forced	Consented	695 (60.4)	Ref	Ref	0.003
		Tricked/Pressured	194 (71.4)	1.64 (1.16–2.31)	1.40 (0.97–2.03)	
		Forced	110 (80.2)	2.65 (1.63–4.31)	1.97 (1.18–3.29)	
Model 3A	Current Intimate Partner	No	392 (59.6)	Ref	Ref	0.0006
		Yes	610 (68.4)	1.47 (1.13–1.90)	1.67 (1.25–2.23)	
	Number of people dependent on her income	0–1	178 (54.3)	Ref	Ref	0.07
		2–3	475 (63.9)	1.49 (1.05–2.12)	1.31 (0.88–1.95)	
		4+	350 (70.7)	2.03 (1.40–2.96)	1.52 (0.98–2.34)	
	Additional income from sex work	No	571 (68.4)	Ref	Ref	0.004
		Yes	432 (60.4)	0.71 (0.55–0.91)	0.65 (0.49–0.87)	
	Recent hunger past 7 days	No	670 (59.6)	Ref	Ref	0.04
		Yes	331 (75.0)	2.03 (1.52, 2.70)	1.39 (1.01, 1.92)	
Model 3B	Street-based sex work	No	705 (62.2)	Ref	Ref	0.08
		Yes	294 (71.5)	1.52 (1.14–2.04)	1.34 (0.97–1.85)	
	Condom use last sex (any partner)	No	236(70.7)	Ref	Ref	0.04
		Yes	765 (63.1)	0.71 (0.52–0.97)	0.68 (0.48, 0.98)	
	Current Prep, PEP or ARV use	No	540 (65.0)	Ref	Ref	0.8
		Yes	450 (65.3)	1.02 (0.79–1.32)	1.04 (0.77–1.40)	
	Police Arrest past 6 months	No	701 (58.0)	Ref	Ref	<0.001
		Yes	302 (80.4)	2.96 (2.16–4.06)	2.40 (1.71–3.39)	
Model 3C	Depression or Anxiety score (PHQ-9/GAD-7)	Low	481 (58.2)	Ref	Ref	0.5
		Mild	279 (67.9)	1.51 (1.11–2.06)	1.26 (0.89–1.78)	
		Moderate/Severe	243 (74.1)	2.05 (1.47–2.87)	1.09 (0.73–1.62)	
	Alcohol risk score (WHO ASSIST)	Low 0–10	697 (59.2)	Ref	Ref	<0.001
		Moderate 11–26	207 (73.6)	1.92 (1.37–2.71)	1.37 (0.94–2.01)	
		High 27+	95 (88.1)	5.11 (2.77–9.42)	3.34 (1.74–6.42)	
	Social support	Not CBO member	907 (63.4)	Ref	Ref	0.01
		Belongs to CBO	96 (78.6)	2.12 (1.30–3.48)	1.91 (1.14–3.21)	

* Proportion with the outcome ** For comparison, the same variables were adjusted for each of the three violence outcome analyses (any recent physical or sexual violence; any recent physical or sexual violence by an intimate partner; any recent physical or sexual violence by a non-intimate partner). Model 1 models adjusted for age and number of ACEs. Model 2 models adjusted for level 1 variables and sexual debut. Model 3a models were adjusted for level 1 and 2 variables, along with level 3a variables shown in the Table. Model 3b models were adjusted for level 1 and 2 variables, along with level 3b variables shown in the Table. Model 3c models were adjusted for level 1 and 2 variables, along with level 3c variables shown in the Table. *** Adjusted Wald Test. Where variables were ordered categorically, we show the Test for Trend.

## Data Availability

The data that support the findings of this study will be available on request from the corresponding author from June 2023 (two years after the study data collection is completed). The data are not publicly available due to privacy or ethical restrictions.

## Supplementary Materials

The following supporting information can be downloaded at: https://www.mdpi.com/article/10.3390/ijerph20116046/s1, Table S1: Multivariable logistic regression—associations with recent physical or sexual violence by an Intimate Partner; Table S2: Multivariable logistic regression—associations with recent physical or sexual violence by a non-Intimate Partner.
