# Supplementary material for: Violence across the Life Course and Implications for Intervention Design: Findings from the Maisha Fiti Study with Female Sex Workers in Nairobi, Kenya
Source: Int J Environ Res Public Health. 2023 Jun 3;20(11):6046. doi: 10.3390/ijerph20116046 (PMC10253020; doi:10.3390/ijerph20116046)
Supplement: Supplementary file 1 [file ijerph-20-06046-s001.zip › ijerph-2182157-supplementary.pdf]

Table S1. Multivariable logistic regression – associations with recent physical or sexual violence by an Intimate Partner

			N (%*)	Crude Odds Ratio (95% CI)	Adjusted Odds Ratio** (95% CI)	P-value***
Model 1	Age (years)	18-24	212 (31.1)	Ref	Ref	0.2
		25-34	353 (34.6)	1.17 (0.82-1.67)	1.20 (0.83-1.73)	
		35-45	438 (28.5)	0.88 (0.62-1.25)	0.90 (0.63-1.31)	
	Total number of Adverse Childhood Experiences	0-4	282 (20.8)	Ref	Ref	<0.001
		5-8	548 (32.8)	1.85 (1.33-2.59)	1.78 (1.26--2.50)	
		9-12	173 (43.6)	2.94 (1.95-4.44)	2.79 (1.83-4.27)	
Model 2	Sexual debut forced	Consented	695 (30.1)	Ref	Ref	0.6
		Tricked/Pressured	194 (31.4)	1.07 (0.76-1.50)	0.93 (0.65-1.33)	
		Forced	110 (37.7)	1.41 (0.93-2.12)	1.17 (0.77-1.79)	
Model 3A	Current Intimate Partner	No	392 (8.6)	Ref	Ref	<0.001
		Yes	610 (46.3)	9.14 (6.25-13.38)	10.12 (6.71-15.25)	
	Number of people dependent on her income	0-1	178 (25.7)	Ref	Ref	0.5
		2-3	475 (32.6)	1.40 (0.95-2.05)	1.13 (0.71-1.81)	
		4+	350 (31.7)	1.34 (0.90-2.01)	0.93 (0.57-1.52)	
	Additional income to sex work	No	571 (27.3)	Ref	Ref	0.07
		Yes	432 (36.3)	1.52 (1.16-1.98)	1.32 (0.97-1.78)	
	Recent hunger past 7 days	No	670 (29.0)	Ref	Ref	0.1
		Yes	331 (35.4)	1.35 (1.02-1.77)	1.31 (0.94-1.82)	
Model 3B	Street based sex work	No	705 (31.0)	Ref	Ref	0.8
		Yes	294 (31.7)	1.03 (0.78-1.38)	0.96 (0.71-1.31)	
	Condom use last sex (any partner)	No	236 (41.6)	Ref	Ref	0.001
		Yes	765 (28.1)	0.55 (0.41-0.74)	0.58 (0.42-0.80)	

	Current Prep, PEP or ARV use	No	540 (35.5)	Ref	Ref	0.006
		Yes	450 (26.7)	0.66 (0.51-0.87)	0.67 (0.50-0.89)	
	Police Arrest past 6 months	No	701 (28.5)	Ref	Ref	0.03
		Yes	302 (37.5)	1.51 (1.14-1.99)	1.43 (1.05-1.95)	
Model 3C	Depression or Anxiety score (PHQ-9 / GAD-7)	Low	481 (22.4)	Ref	Ref	0.02
		Mild	279 (41.1)	2.42 (1.77-3.33)	2.22 (1.58-3.12)	
		Moderate/Severe	243 (36.8)	2.02 (1.45-2.82)	1.40 (0.95-2.04)	
	Alcohol risk score (WHO ASSIST)	Low 0-10	697 (25.9)	Ref	Ref	0.001
		Moderate 11-26	207 (40.7)	1.96 (1.42-2.71)	1.50 (1.05-2.14)	
		High 27+	95 (48.8)	2.73 (1.77-4.21)	2.06 (1.28-2.32)	
	Social support	Not CBO member	907 (30.3)	Ref	Ref	0.1
		Belongs to CBO	96 (39.9)	1.53 (1.00-2.35)	1.46 (0.92-2.33)	

*Proportion with the outcome ** For comparison, the same variables were adjusted for in each of the three violence outcome analyses (any recent physical or sexual violence; any physical or sexual violence by an intimate partner; any recent physical or sexual violence by a non-intimate partner. Model 1 models adjusted for age, and number of ACEs. Model 2 models adjusted for level 1 variables, and sexual debut. Model 3a models were adjusted for level 1 and 2 variables, along with level 3a variables shown in the Table. Model 3b models were adjusted for level 1 and 2 variables, along with level 3b variables shown in the Table. Model 3c models were adjusted for level 1 and 2 variables, along with level 3c variables shown in the Table. ***Adjusted Wald Test. Where variables were ordered categorical, we show the Test for Trend.

Table S2. Multivariable logistic regression – associations with recent physical or sexual violence by a non-Intimate Partner

			N (%*)	Crude Odds Ratio (95% CI)	Adjusted Odds Ratio** (95% CI)	P-value***
Model 1	Age (years)	18-24	212 (51.4)	Ref	Ref	0.6
		25-34	353 (58.9)	1.36 (0.97-1.90)	1.48 (1.03-2.14)	
		35-45	438 (54.1)	1.11 (0.81-1.54)	1.31 (0.91-1.88)	
	Total number of Adverse Childhood Experiences	0-4	282 (32.6)	Ref	Ref	<0.001
		5-8	548 (60.5)	3.17 (2.35-4.28)	3.15 (2.33-4.26)	
		9-12	173 (78.7)	7.67 (4.98-11.82)	7.48 (4.82-11.62)	
Model 2	Sexual debut forced	Consented	695 (49.8)	Ref	Ref	<0.001
		Tricked/Pressured	194 (64.3)	1.82 (1.31-2.52)	1.54 (1.10-2.17)	
		Forced	110 (75.8)	3.17 (2.01-4.99)	2.46 (1.51-4.00)	
Model 3A	Current Intimate Partner	No	392 (57.9)	Ref	Ref	0.5
		Yes	610 (54.1)	0.86 (0.67-1.10)	0.91 (0.69-1.21)	
	Number of people dependent on her income	0-1	178 (46.0)	Ref	Ref	0.02
		2-3	475 (53.0)	1.32 (0.94-1.87)	1.20 (0.80-1.78)	
		4+	350 (63.5)	2.04 (1.41-2.95)	1.59 (1.04-2.43)	
	Additional income to sex work	No	571 (61.8)	Ref	Ref	<0.001
		Yes	432 (47.8)	0.56 (0.44-0.73)	0.53 (0.40-0.70)	
	Recent hunger past 7 days	No	670 (49.2)	Ref	Ref	0.01
		Yes	331 (68.5)	2.25 (1.71-2.96)	1.43 (1.08- 1.96)	
Model 3B	Street based sex work	No	705 (52.4)	Ref	Ref	0.01
		Yes	294 (63.8)	1.60 (1.22-2.11)	1.48 (1.08-2.01)	
	Condom use last sex (any partner)	No	236 (58.7)	Ref	Ref	0.3
		Yes	765 (54.7)	0.85 (0.63-1.14)	0.83 (0.59-1.17)	

	Current Prep, PEP or ARV use	No	540 (53.3)	Ref	Ref	0.1
		Yes	450 (58.3)	1.22 (0.95-1.56)	1.24 (0.93-1.66)	
	Police Arrest past 6 months	No	701 (48.1)	Ref	Ref	<0.001
		Yes	302 (72.9)	2.91 (2.17-3.88)	2.32 (1.69-3.17)	
Model 3C	Depression or Anxiety score (PHQ-9 / GAD-7)	Low	481 (49.8)	Ref	Ref	0.6
		Mild	279 (53.1)	1.14 (0.85-1.53)	0.90 (0.64-1.26)	
		Moderate/Severe	243 (69.6)	2.31 (1.67-3.19)	1.17 (0.80-1.72)	
	Alcohol risk score (WHO ASSIST)	Low 0-10	697 (49.6)	Ref	Ref	<0.001
		Moderate 11-26	207 (63.5)	1.77 (1.29-2.43)	1.33 (0.93-1.90)	
		High 27+	95 (84.6)	5.57 (3.21-9.66)	3.92 (2.19-6.99)	
	Social support	Not CBO member	907 (54.3)	Ref	Ref	0.03
		Belongs to CBO	96 (68.8)	1.86 (1.19-2.89)	1.66 (1.04-2.64)	

*Proportion with the outcome ** For comparison, the same variables were adjusted for in each of the three violence outcome analyses (any recent physical or sexual violence; any physical or sexual violence by an intimate partner; any recent physical or sexual violence by a non-intimate partner. Model 1 models adjusted for age, and number of ACEs. Model 2 models adjusted for level 1 variables, and sexual debut. Model 3a models were adjusted for level 1 and 2 variables, along with level 3a variables shown in the Table. Model 3b models were adjusted for level 1 and 2 variables, along with level 3b variables shown in the Table. Model 3c models were adjusted for level 1 and 2 variables, along with level 3c variables shown in the Table. ***Adjusted Wald Test. Where variables were ordered categorical, we show the Test for Trend.
